# Supplementary material for: Radionuclide tracing based in situ corrosion and mass transport monitoring of 316L stainless steel in a molten salt closed loop
Source: Nat Commun. 2024 Apr 10;15:3106. doi: 10.1038/s41467-024-47259-8 (PMC11271644; doi:10.1038/s41467-024-47259-8)
Supplement: Supplementary file 1 — Supplementary information [file 41467_2024_47259_MOESM1_ESM.pdf]

Supplementary Information

Radionuclide Tracing Based *in situ* Corrosion and Mass Transport Monitoring of 316L Stainless Steel in a Molten Salt Closed Loop

Yafei Wang[1,2,*], Aeli P. Olson[3], Cody Falconer[4,5], Brian Kelleher[5], Ivan Mitchell[5], Hongliang Zhang[4], Kumar Sridharan[1,4], Jonathan W. Engle[3], Adrien Couet[1,4]

[1]Department of Engineering Physics, University of Wisconsin-Madison, WI 53706, USA

[2]School of Nuclear Science and Engineering, Shanghai Jiao Tong University, Shanghai 200240, China

[3]Departments of Medical Physics and Radiology, University of Wisconsin-Madison, WI 53705, USA

[4]Department of Materials Science and Engineering, University of Wisconsin-Madison, WI 53706, USA

[5]TerraPower, LLC, Bellevue, WA98008, USA

*itsme@sjtu.edu.cn

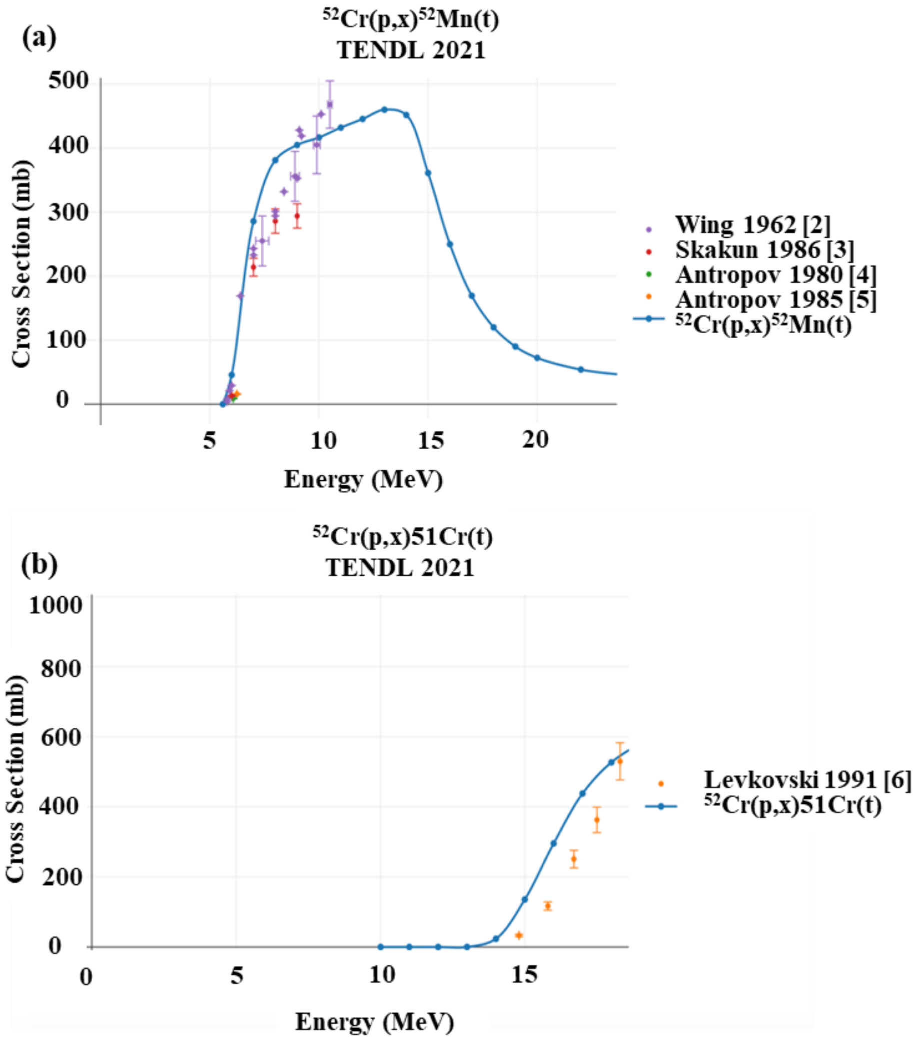

Supplementary Fig. 1. **Reaction cross-sections for different radionuclides at different energies.** (a) ^{52}Mn and (b) ^{51}Cr. [1]. Error bar is the data uncertainty originating from the gamma spectra instrumental measurement sensitivity.

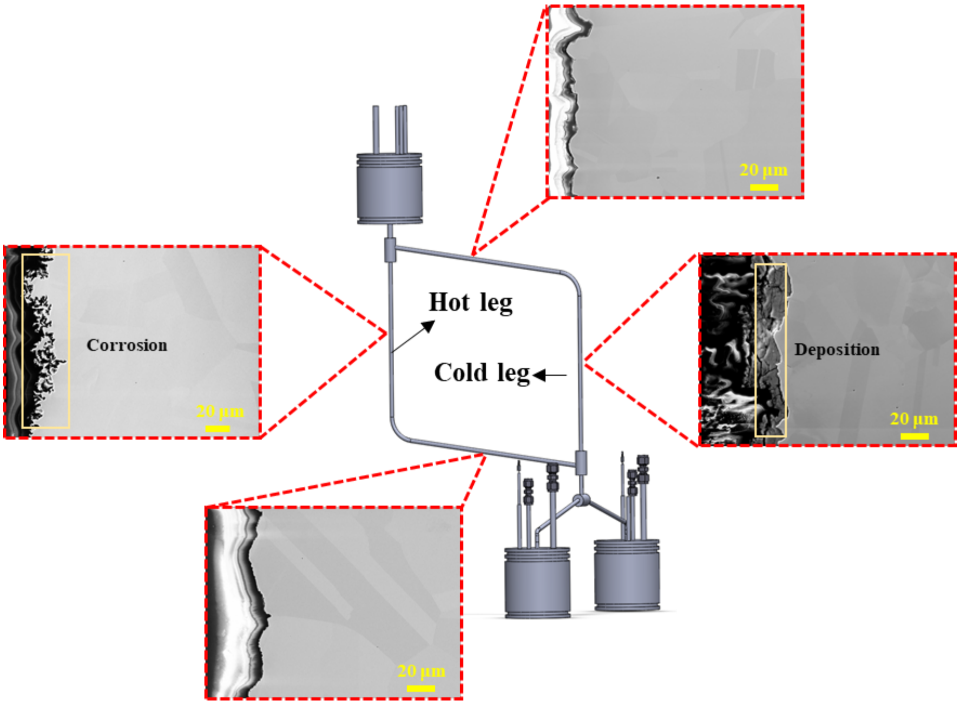

Supplementary Fig. 2. **Material characterization of different tube sections along the loop.** Hot leg is severely corroded; cold leg has corrosion products deposited; the top and bottom sections of loop are barely corroded.

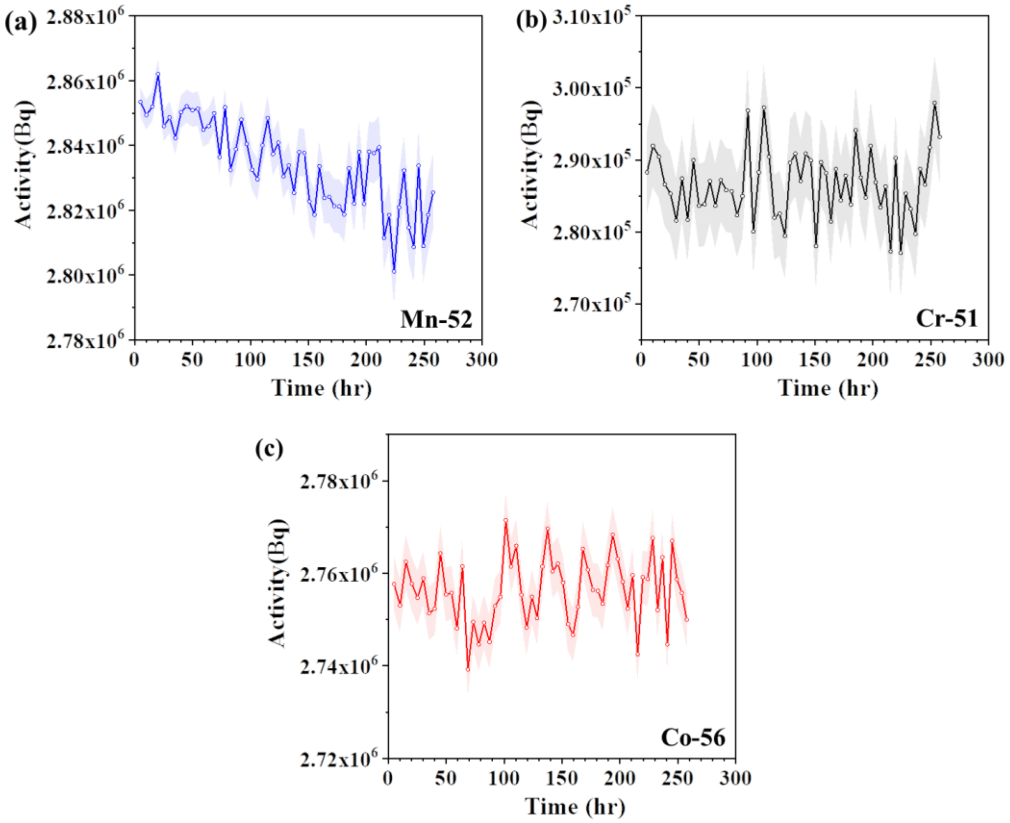

Supplementary Fig. 3. **Activity variations of radionuclides with time during the loop operation process.** (a) ^{52}Mn, (b) ^{51}Cr, and (c) ^{56}Co. The shaded area is for data uncertainty sourced from counting statistical calculation.

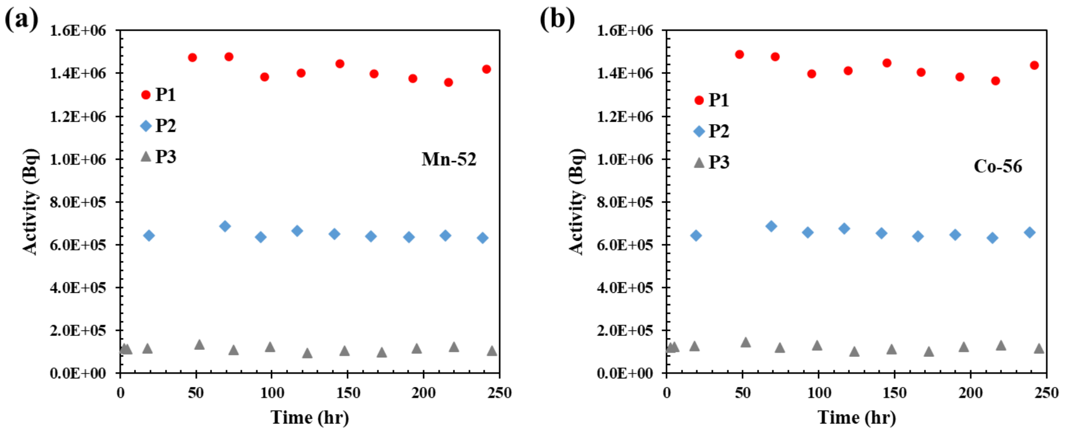

Supplementary Fig. 4. **Activity variations at three different locations along the loop during its operation.** (a) ^{52}Mn and (b) ^{56}Co. Data uncertainty sourced from counting statistical calculation is not visible due to the large y-axis scale.

Supplementary Table 1. Nominal chemical composition of 316L SS (in wt%, Bal. Fe).

Element	C	Mn	P	S	Si	Cr	Ni	Mo
Composition	0.035	2.00	0.04	0.03	1.00	16.0~18.0	10.0~15.0	2.00~3.00

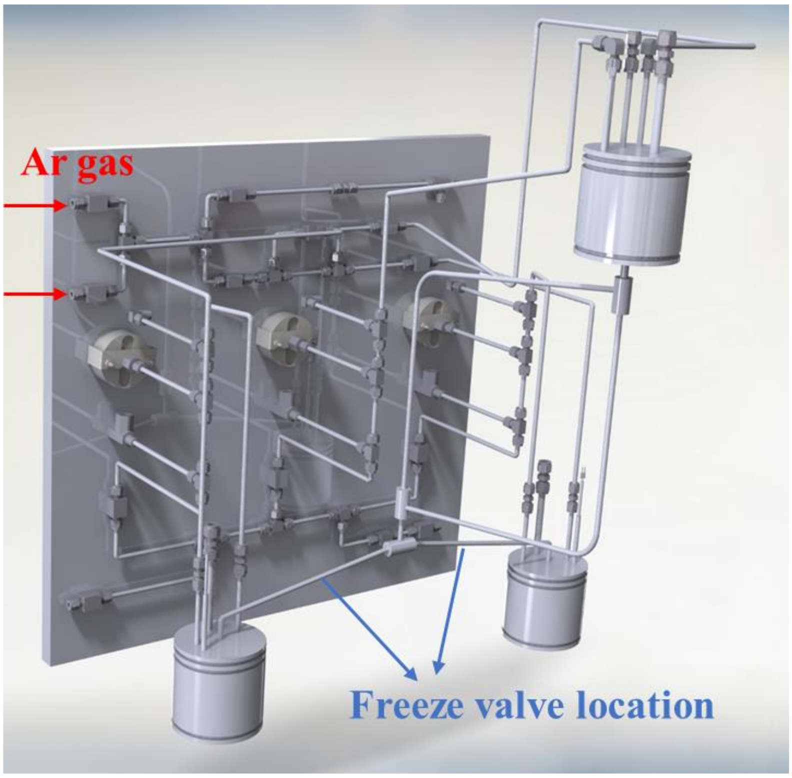

Supplementary Fig. 5. **The Schematic of the molten salt microloop.** The loop body itself is connected with the auxiliary parts.

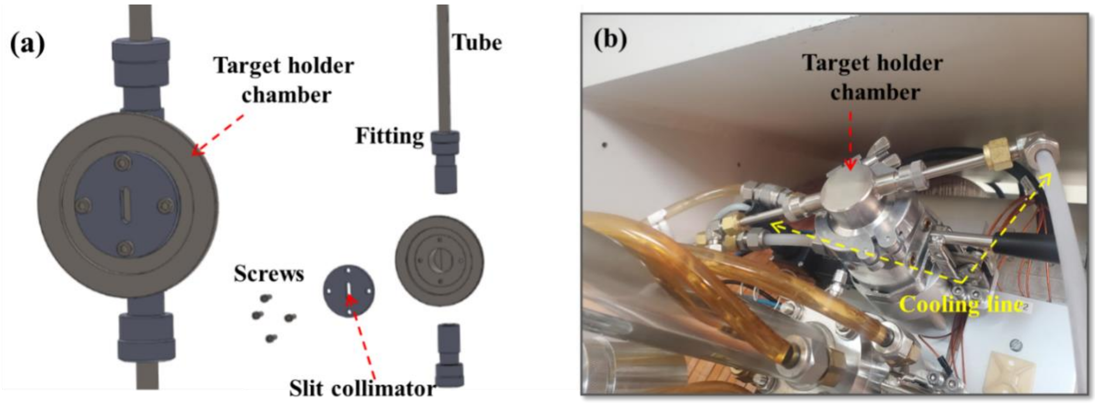

Supplementary Fig. 6. **Irradiation experiment setup.** (a) Schematic illustration of the designed target holder and slit collimator for tube irradiation. (b) Target holder in cyclotron GE PETtrace before irradiation.

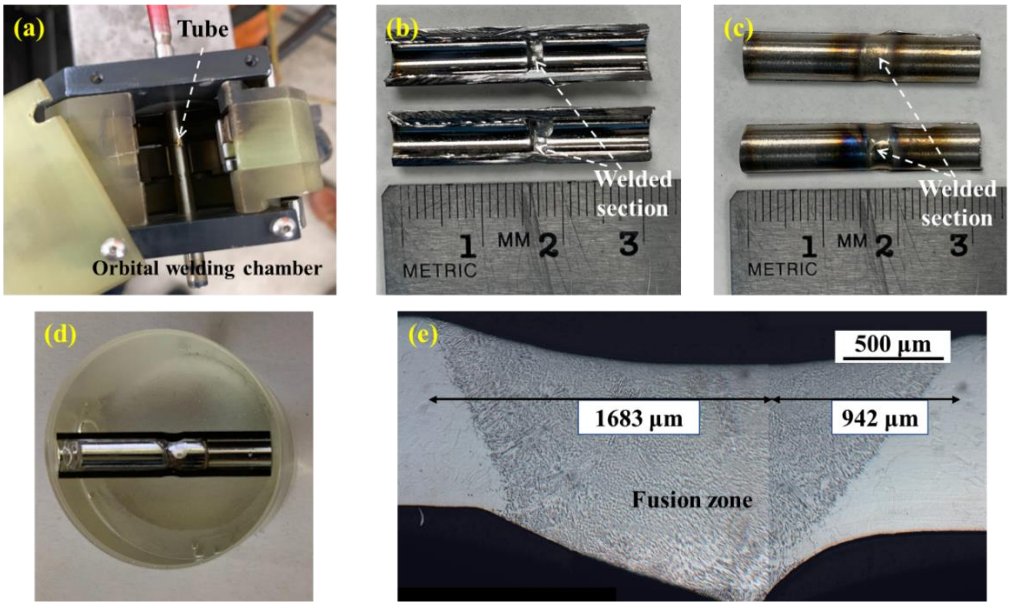

Supplementary Fig. 7. **Orbital welding of 316L SS tube.** (a) Orbital welder chamber for the welding of 316L SS tubes. (b) Inside view of the welded tube after being cut axially. (c) Outside view of the welded tube after being cut axially. (d) Cross section of the welded tube after being mounted and polished. (e) Heat affected/fusion zone of the orbital welding on two 316L SS tubes.

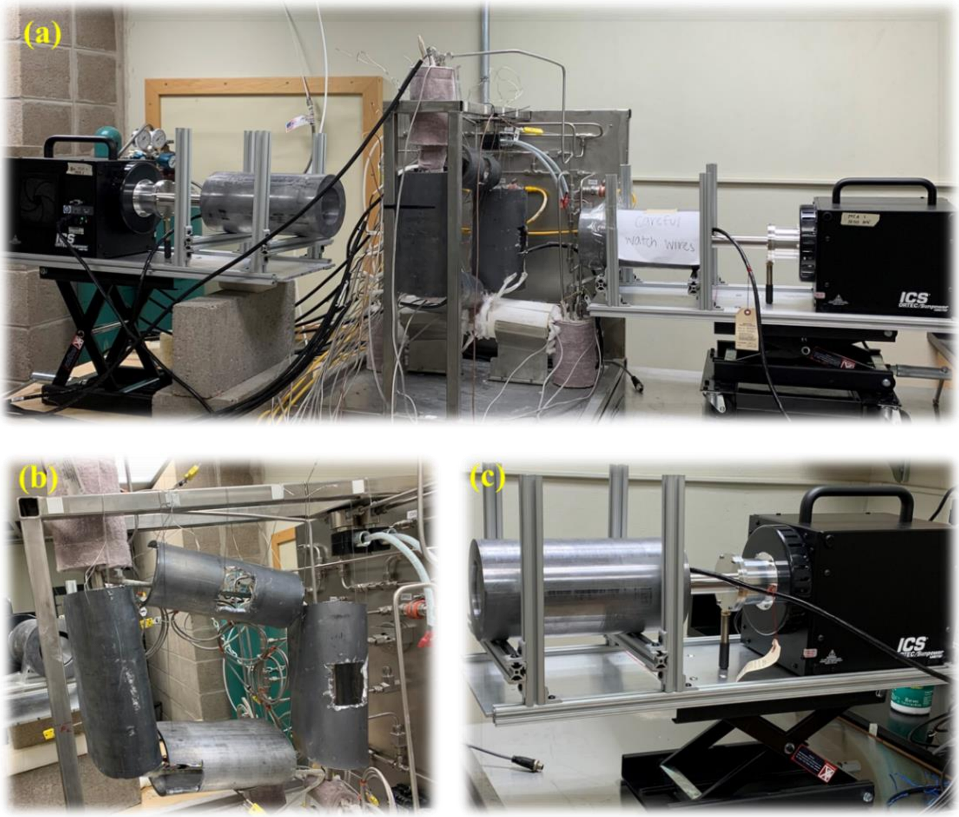

Supplementary Fig. 8. ***In situ* corrosion testing setup.** (a) Microloop *in situ* corrosion monitoring system. (b) Lead shielding for the microloop. (c) Cylindrical lead shielding for the HPGe detector.

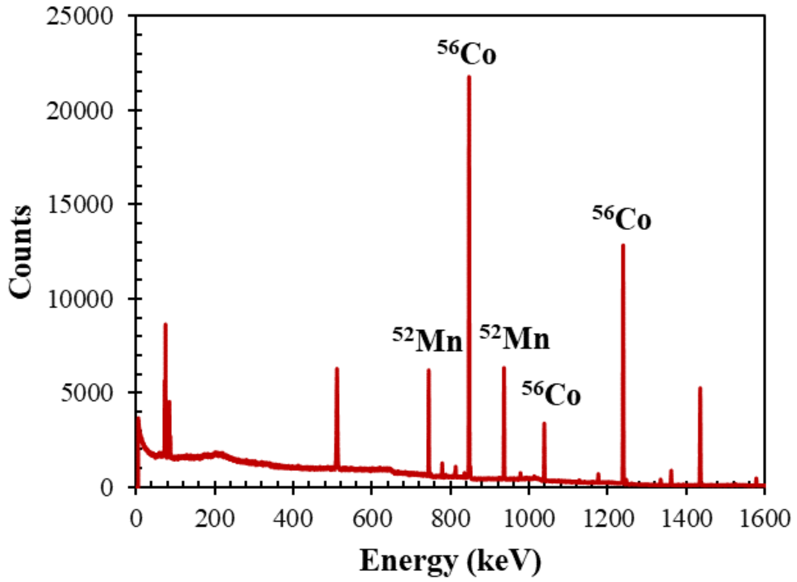

Supplementary Fig. 9. **Gamma-ray spectrum obtained at the cold leg of the loop before corrosion testing with lead shield.** The energy peaks of ^{52}Mn and ^{56}Co have been labeled; no ^{51}Cr is detected.

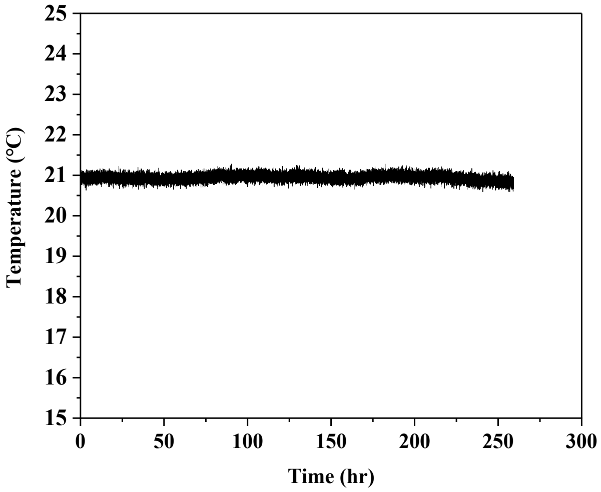

Supplementary Fig. 10. **Room temperature variation during the corrosion testing.** Temperature is stable over through the whole testing period.

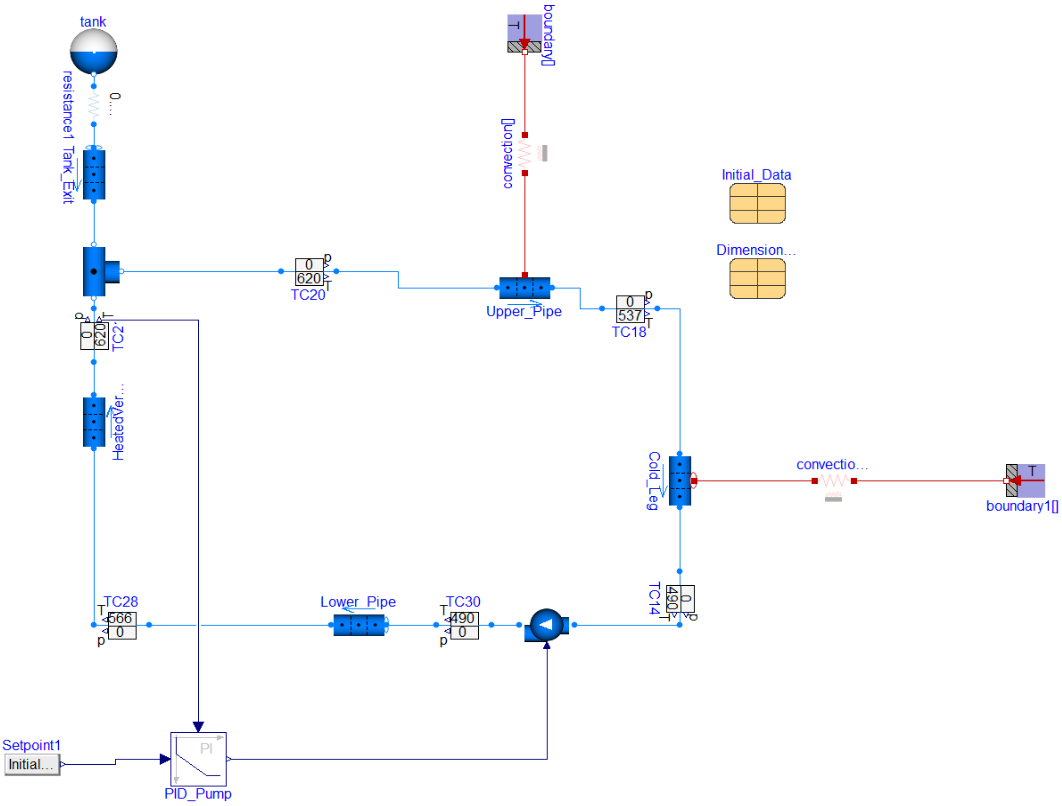

Supplementary Fig. 11. **Schematic diagram of the TRANSFORM model of the natural circulation microloop.** Temperature data from experiments are used as the input vectors.

(a)

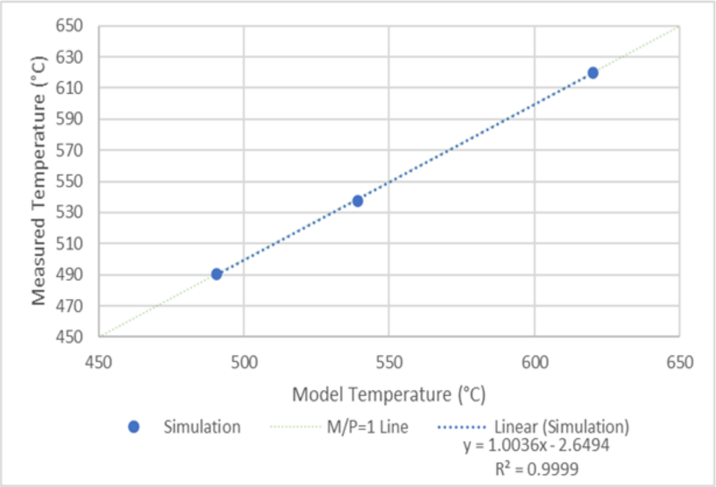

(b)

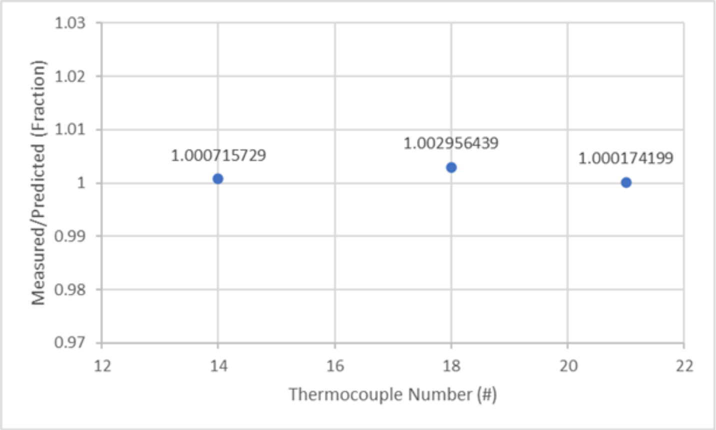

Supplementary Fig. 12. **The comparison of the predicted and measured thermocouple reading in the microloop by TRANSFORM model.** (a) Measured vs predicted temperatures; (b) ratio of measured and predicted temperature.

Supplementary References

[1] Koning, Arjan, Rochman, Dimitri A., Sublet, J. Ch, Dzysiuk, Nataliya R., Fleming, Michael J., and van der Mark, Steven C. TENDL: Complete Nuclear Data Library for Innovative Nuclear Science and Technology. Nucl. Data Sheets, 155, 1-55 (2019).

[2] Wing, J., and J. R. Huizenga. (p, n) Cross Sections of V 51, Cr 52, Cu 63, Cu 65, Ag 107, Ag 109, Cd 111, Cd 114, and La 139 from 5 to 10.5 MeV. Physical Review 128 (1), 280, (1962).

[3] Skakun, E. A., V. G. Batij, Ju N. Rakivnenko, and O. A. Rastrepin. Investigation of cross sections of cr-52 (p, n) mn-52-m, g and cr-54 (p, n) mn-54 reactions in the energy range from 5 to 9 mev. In Conf. nucl. spectrosc. and nucl. struct., kharkov, 277, (1986).

[4] Antropov AE, Gusev VP, Zarubin PP, Ioannu PD, Padalko VYu. Measurement of the total cross section for the (p, n) reaction on medium mass atomic nuclei with 6 MeV protons. 30th Conference nuclear spectroscopy and nuclear structure, USSR, Leningrad, 316, EXFOR: A0072, (1980)

[5] Antropov AE, Zarubin PP, Aleksandrov Yu A, Gorshkov IY. Study of the cross

section for the reactions (p, n), (α, pn),(α, xn) on medium weight nuclei. In: 35th Conference nuclear spectroscopy and nuclear structure, Leningrad, 369. EXFOR: O0076, (1985).
[6] Levkovskij, V. N. Activation cross section nuclides of average masses (A= 40–100) by protons and alpha-particles with average energies (E= 10–50 MeV). Moscow, Russia, (1991).
